# VIPP1 rods engulf membranes containing phosphatidylinositol phosphates

**DOI:** 10.1038/s41598-019-44259-3

**Published:** 2019-06-19

**Authors:** Jasmine Theis, Tilak Kumar Gupta, Johannes Klingler, William Wan, Sahradha Albert, Sandro Keller, Benjamin D. Engel, Michael Schroda

**Affiliations:** 1 Molecular Biotechnology and Systems Biology, Technische Universität Kaiserslautern (TUK), Paul-Ehrlich Straße 23, 67663 Kaiserslautern, Germany; 2 Department of Molecular Structural Biology, Max-Planck-Institute of Biochemistry, Am Klopferspitz 18, 82152 Martinsried, Germany; 3 Molecular Biophysics, Technische Universität Kaiserslautern (TUK), Erwin-Schrödinger-Str. 13, 67663 Kaiserslautern, Germany

**Keywords:** Photosynthesis, Chloroplasts

## Abstract

In cyanobacteria and plants, VIPP1 plays crucial roles in the biogenesis and repair of thylakoid membrane protein complexes and in coping with chloroplast membrane stress. In chloroplasts, VIPP1 localizes in distinct patterns at or close to envelope and thylakoid membranes. *In vitro*, VIPP1 forms higher-order oligomers of >1 MDa that organize into rings and rods. However, it remains unknown how VIPP1 oligomerization is related to function. Using time-resolved fluorescence anisotropy and sucrose density gradient centrifugation, we show here that *Chlamydomonas reinhardtii* VIPP1 binds strongly to liposomal membranes containing phosphatidylinositol-4-phosphate (PI4P). Cryo-electron tomography reveals that VIPP1 oligomerizes into rods that can engulf liposomal membranes containing PI4P. These findings place VIPP1 into a group of membrane-shaping proteins including epsin and BAR domain proteins. Moreover, they point to a potential role of phosphatidylinositols in directing the shaping of chloroplast membranes.

## Introduction

VIPP1 is a highly conserved protein found in cyanobacteria and the chloroplasts of both algae and land plants. VIPP1 was originally proposed to be involved in the biogenesis of the lipid portion of thylakoid membranes by playing a role in vesicular traffic, which led to its naming as vesicle-inducing protein in plastids^1–3^. This specific role in vesicle formation has been questioned, and VIPP1 is now believed to play roles in coping with chloroplast membrane stress^4–6^ and assisting the biogenesis and repair of thylakoid protein complexes^7–14^. VIPP1 evolved from the bacterial phage shock protein A (PspA), with which it shares several structural features: both proteins consist of *α*-helical domains connected by random-coil spacers, but VIPP1 possesses an additional domain of about 30 amino acids at its C-terminus^2,15^. Moreover, both proteins have a 24 amino acid stretch at their N-terminus that forms an amphipathic α-helix (AHa) and is required for membrane binding and the formation of larger oligomers^15–19^. *In vitro*, both proteins form higher-order oligomers of >1 MDa that give rise to rings and rods^20–25^. In chloroplasts, VIPP1 accumulates at or close to envelope and thylakoid membranes, producing a variety of localization patterns including dots, lines, forks, and webs^4,5,11,21,26^. It was recently proposed that the AHa of PspA evolved specifically to bind stressed membranes, while the AHa of VIPP1 could selectively target areas with elevated levels of anionic lipids, which might be the key to its role in the biogenesis and repair of thylakoid membrane protein complexes^19^. In this study, we have addressed the lipid specificity of *Chlamydomonas reinhardtii* VIPP1. We show that this protein binds strongly to phosphatidylinositol phosphates (PIPs) and that rods formed by VIPP1 oligomers can engulf liposomes containing phosphatidylinositol-4-phosphate (PI4P).

## Results and Discussion

### Chlamydomonas VIPP1 binds strongly to phosphatidylinositol phosphates

**Figure 1:**
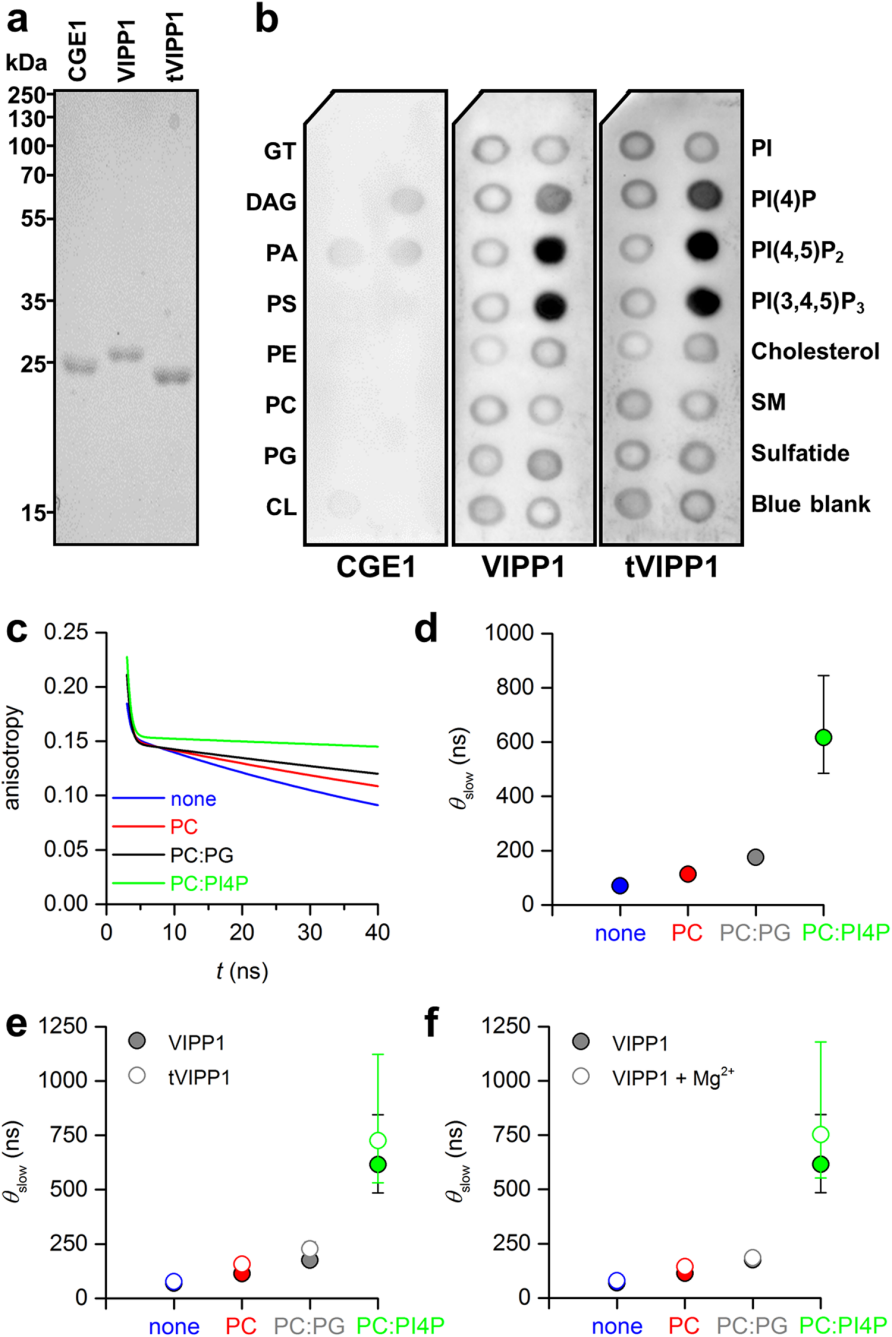
The sizes of VIPP1-containing particles increase in the presence of PC:PI4P- liposomes. (**a**) Separation of 2 µg each of recombinant VIPP1, tVIPP1 and CGE1 on a 12% SDS-polyacrylamide gel, stained with Coomassie brilliant blue. (**b**) Protein–lipid overlay assay. Membranes spotted with different lipid species were incubated with the recombinant proteins followed by immunodetection of the bound proteins. Lipid species spotted were GT – glyceryl tripalmitate, DAG – diacylglycerol, PA – phosphatidic acid, PS – phosphatidylserine, PE – phosphatidylethanolamine, PC – phosphatidylcholine, PG – phosphatidylglycerol, CL – cardiolipin, PI – phosphatidylinositol, PIP – phosphatidylinositol phosphate, SM – sphingomyelin. Blue blank (Xylene Cyanol FF) is the negative control. (**c**) Fitted fluorescence anisotropy decay curves for VIPP1 without liposomes (none) and mixtures of VIPP1 with liposomes composed of PC, PC:PG (95:5), and PC:PI4P (95:5). (**d**) Best-fit values and 68% confidence intervals for the slow correlation times, *θ*_slow_, which reflect the rotational mobility of VIPP1. Note that in most cases error bars are too small to be seen. (**e**) Best-fit values and 68% confidence intervals for *θ*_slow_ for the measurement shown in d and a measurement with tVIPP1. (**f**) Best-fit values and 68% confidence intervals for *θ*_slow_ for the measurement shown in d and a subsequent measurement performed in the presence of 10 mM Mg^2+^.

Cyanobacterial VIPP1 binds to liposomes containing the anionic lipids phosphatidylglycerol (PG) and sulfoquinovosyldiacylglycerol (SQDG) but not to liposomes composed of only neutral lipids^27^. To test whether this is also true for chloroplast VIPP1, we recombinantly produced VIPP1 from *Chlamydomonas reinhardtii*. We also produced a VIPP1 variant lacking the C-terminal extension that distinguishes VIPPs from bacterial PspAs^2^ (tVIPP1; VIPP1 missing amino acids 220–251). As a control, we used the chloroplast GrpE homolog (CGE1), a stromal protein that, like VIPP1, has a high α-helical content^28^ (Fig. 1a). Upon subjecting the three recombinant proteins to a lipid-overlay assay, we observed a strong interaction of VIPP1 and tVIPP1 with phosphatidylinositol phosphates (PIPs), but not with any of the other lipids on the membrane, including PG (Fig. 1b).

To substantiate this finding, we generated liposomes composed of the neutral lipid phosphatidylcholine (PC) and PI4P at a molar ratio of 95:5, as phosphatidylinositols typically represent 1–5% of the lipids in the envelopes and thylakoids of algal and higher-plant chloroplasts^29,30^. Integration of PI4P into the liposomes was verified by ^31^P NMR spectroscopy (Supplementary Fig. 1). To monitor the binding of VIPP1 to PC:PI4P liposomes, we took advantage of the fact that *Chlamydomonas* VIPP1 has two tryptophan residues, which we probed by time-resolved fluorescence (TRF) anisotropy. As shown in Fig. 1c,d, the addition of PC:PI4P liposomes considerably slowed down the rotational dynamics of the tryptophan residues in VIPP1. By contrast, pure PC liposomes and PC:PG (95:5) liposomes had only minor effects. The slowed rotational dynamics in the presence of PC:PI4P liposomes reflect an increase in overall particle size, motional restrictions within VIPP1 monomer units upon lipid binding, or a combination of these two effects. Both are indicative of VIPP1 interactions with liposomal lipids. Very similar results were obtained for tVIPP1 (Fig. 1e), indicating that the observed interaction of VIPP1 with PC:PI4P liposomes did not depend on the VIPP1 C-terminus. Cyanobacterial VIPP1 together with Mg^2+^ at concentrations above 5 mM was previously found to destabilize liposome membranes, thereby facilitating liposome fusion^27^. This effect was attributed to a reorganization of VIPP1 protein structure upon Mg^2+^ binding, resulting in increased exposure of hydrophobic surfaces and ring stacking^31^. Moreover, VIPP1 from *Arabidopsis thaliana* was found to exhibit Mg^2+^-dependent GTPase activity^32^. We therefore also monitored the interaction of VIPP1 with liposomes consisting of PC, PC:PG, and PC:PI4P in the presence of 10 mM Mg^2+^. As shown in Fig. 1f, Mg^2+^ ions had no significant effect on the interaction of *Chlamydomonas *VIPP1 with any of the three liposome types.

To verify the interaction of VIPP1 to PC:PI4P liposomes by a complementary method, we subjected recombinant VIPP1 alone or VIPP1 in the presence of pure PC liposomes or PC:PI4P liposomes to rate-zonal centrifugation on sucrose gradients (Supplementary Fig. 2). VIPP1 alone was detected almost exclusively on the top half of the gradient, while only a minor fraction of VIPP1 was detected in the lower half of the gradient run with VIPP1 and pure PC liposomes. Strikingly, in the gradient run with VIPP1 and PC:PI4P liposomes, most of the VIPP1 was found in the bottom fraction, indicating the formation of larger particles upon binding of VIPP1 to PC:PI4P liposomes.

### Chlamydomonas VIPP1 forms rods that encapsulate lipids

**Figure 2:**
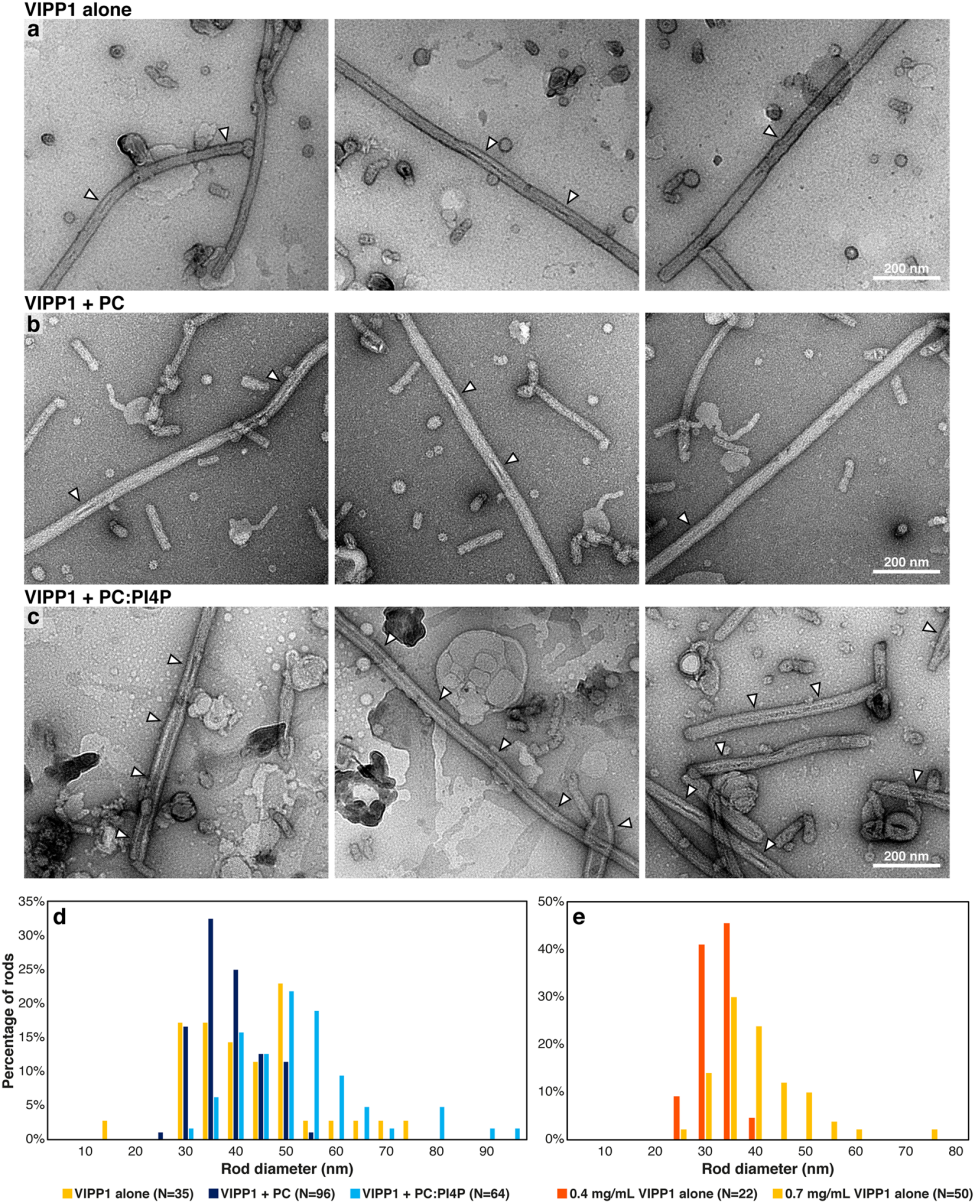
VIPP1 forms large rods that can contain lipids. Negative stain electron micrographs of (**a**) VIPP1 alone, (**b**) VIPP1 incubated with PC liposomes and (**c**) VIPP1 incubated with PC:PI4P (95:5) liposomes. The same VIPP1 preparation (0.4 mg/mL, Prep #4, see methods) was used for all conditions, and this sample was also used for the “VIPP1 + PC:PI4P” cryo-ET in Figs. 3 and 4 (see methods). Encapsulated distinctly stained material inside the VIPP1 rods is indicated with arrowheads. (**d**) Distribution of rod diameters for the three conditions in (**a**–**c)**. (**e**) Distribution of rod diameters for VIPP1 alone for two different VIPP1 preparations at concentrations of 0.4 mg/mL and 0.7 mg/mL (Preps #2 and #1, respectively, see methods). The 0.4 mg/mL sample was also used for the “VIPP1 alone” cryo-ET in Figs. 3 and 4.

We wondered what structural changes occur when VIPP1 particles increase in size by interacting with liposomes, so we performed negative-stain electron microscopy of VIPP1 alone, VIPP1 incubated with pure PC liposomes, and VIPP1 incubated with PC:PI4P liposomes (Fig. 2a–c). As observed previously^22^, VIPP1 formed rings and rods under all conditions. Interestingly, we found that distinctly stained material was encapsulated by these rods. Both in the absence of liposomes and when incubated with pure PC liposomes, VIPP1 rods occasionally contained short stretches of such material. By contrast, VIPP1 preparations incubated with PC:PI4P liposomes contained this material over long stretches within the rods. We reasoned that the encapsulated material in the VIPP1 preparations not incubated with liposomes represented endogenous *E*. *coli* lipids, as VIPP1 was purified under native conditions, and *E*. *coli* lipids have previously been observed in recombinant VIPP1 preparations^15^. Pure PC liposomes apparently were encapsulated by VIPP1 rods with low efficiency, whereas PC:PI4P liposomes were encapsulated with high efficiency. Rod diameters only marginally increased in the presence of PC:PI4P liposomes (median of 42 nm for VIPP1 alone vs. 46 nm for VIPP1 + PC:PI4P liposomes; Fig. 2d). This indicates that the distinct size increase seen after incubating VIPP1 with PC:PI4P liposomes in the sucrose density and TRF anisotropy experiments did not result from wider VIPP1 rod diameters. Rod diameters rather appeared to depend on the concentration of VIPP1 protein in the preparation, independent of liposome encapsulation (Fig. 2e).

### Preformed VIPP1 rods can engulf PI4P-containing liposomes, reminiscent of bubbles sucked up into straws

**Figure 3:**
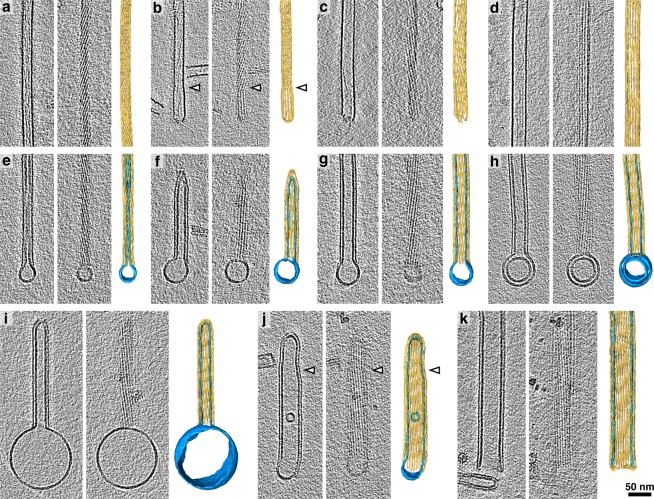
Cryo-electron tomography reveals that VIPP1 rods engulf PI4P-containing liposomes. For each panel, the left image is a slice from the tomogram showing a central longitudinal section through the VIPP1 rod, the middle image is a slice from the tomogram showing either the top or bottom surface of the rod, and the right image is a 3D segmentation of the rod (yellow) and the liposome (blue). (**a–d**) VIPP1 rods alone and (**e–k**) VIPP1 rods incubated with PC:PI4P (95:5) liposomes. The VIPP1 rods shown in (**a–d**) and (**e–k**) were from two different VIPP1 protein preparations (see methods). Both with and without liposomes, the VIPP1 rods exhibit a range of diameters and helical pitches, which can change at discrete points along the rod (arrowheads in b and j, see also Supplementary Fig. 5).

To better understand the molecular architecture of VIPP1 rods with encapsulated lipids, we employed cryo-electron tomography (cryo-ET) on VIPP1 alone and VIPP1 incubated with PC:PI4P liposomes. To our surprise, we found that VIPP1 rods apparently were not composed of stacked rings, as we have previously suspected from negative-stain EM images^22^, but rather represent continuous tubules with striations visible in the surface views (Fig. 3). PC:PI4P liposomes were squeezed inside the hollow VIPP1 rods, regardless of rod diameter, reminiscent of bubbles sucked up into straws (Fig. 3e–k). We never observed an empty rod in this sample; every rod contained a liposome (*N* > 100). As the VIPP1 rods were preassembled prior to liposome addition, the liposomes were likely drawn into the rods by a strong interaction with the rod’s inner surface until the entirety of the rod was filled, leaving the remainder of the liposome attached as a “bubble” on one end of the rod. These cryo-ET observations strongly suggest that the larger size of VIPP1-containing particles upon incubation with PC:PI4P liposomes (Fig. 1) is due to the engulfment of the liposomes. The specific nature of the interaction between the liposome and the inside of the VIPP1 rod can be inferred from Fig. 3h, where only the outer membrane of a bi-lamellar liposome was sucked into the rod. By contrast, the small inner liposome in Fig. 3j was able to enter the rod because it is smaller than the rod’s diameter. Note that such cases were observed only rarely. The VIPP1 concentration used in all of our experiments (0.3 to 0.7 mg/mL, see methods) is close to its physiological concentration: *Chlamydomonas* cells have a volume of ~270 µm^3^ and contain ~20 pg protein^33,34^, leading to a cellular protein concentration of ~74 mg/mL. VIPP1 represents 0.05% of cellular protein^22^, but is only present in the chloroplast stroma, which occupies around one fourth of the cell volume (half of the chloroplast)^34,35^, yielding an estimated concentration of ~0.15 mg/mL VIPP1 in the stroma.

**Figure 4:**
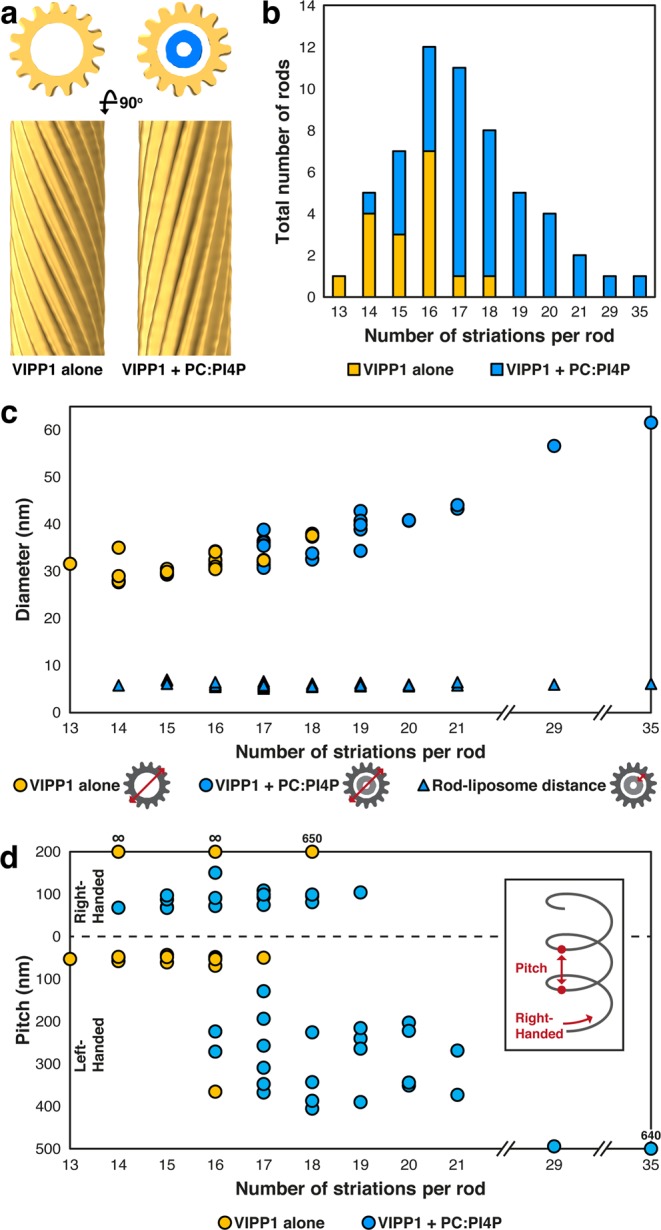
Subtomogram analysis of VIPP1 rods. (**a**) Example subtomogram averages from single VIPP1 rods (yellow) without (left) and with (right) PC:PI4P (95:5) liposomes (blue). Top: cross-sections through averages, bottom: longitudinal views of the rod outer surfaces. For more examples see Supplementary Figs. 3 and 4. (**b**) Distribution of the number of striations per rod in the two cryo-ET datasets, which were acquired from two separate VIPP1 preparations (see methods). (**c**) Plot of VIPP1 rod diameter vs. number of striations (circles) for the two cryo-ET datasets. For the sample containing VIPP1 and PC:PI4P liposomes, the center-to-center distance between the wall of the VIPP1 rod and the membrane bilayer of the engulfed liposome is also plotted (triangles). (**d**) Plot of VIPP1 rod helical pitch vs. number of striations for the two cryo-ET datasets. Both left- and right-handed pitches are plotted. Values are listed above individual points for rods with pitches outside the plotted range. “∞” indicates non-helical rods with striations oriented parallel to the rod’s long axis.

We next performed subtomogram averaging of the cryo-ET dataset (Fig. 4), with the aim of gaining structural insights into the VIPP1–liposome interaction. Unfortunately, the extreme heterogeneity of VIPP1 rod structure (with variable diameter, helical pitch, and handedness) prevented us from combining multiple rods together to generate a high-resolution average of single VIPP1 proteins within the rod. Nonetheless, we were able to generate averages of individual rods (Fig. 4a; Supplementary Figs. 3 and 4), enabling accurate measurement of each rod’s geometric parameters. The number of striations per rod in the two VIPP1 preparations studied was broadly distributed, ranging from 13 to 35 with the maximum of the distribution at 16 striations (Fig. 4b). Rod diameters ranged from ~28 nm up to ~60 nm and increased linearly with striation numbers (Fig. 4c; Supplementary Figs. 3 and 4). The diameter of the engulfed lipid tubule was set by the rod diameter, with the center-to-center distance between the bilayer of the lipid tubule and the wall of the VIPP1 rod remaining at a constant ~5 nm (Fig. 4c). This suggests that VIPP1 rods specifically interacted with lipid membranes instead of randomly enclosing them. Rods of all diameters were capable of engulfing liposomes. The wall thickness of the encapsulated lipid tubule was ~6 nm, consistent with a lipid bilayer.

We observed a higher number of striations, and corresponding wider diameter, for some VIPP1 rods engulfing PC:PI4P lipids compared to empty VIPP1 rods (Fig. 4b,c). While it is tempting to speculate that the engulfed liposomes are responsible for the wider rod diameters, it is important to note that the empty and liposome-encapsulating rods came from different preparations of the VIPP1 protein (see methods). We observed major variations in rod diameter between different preparations, which may be correlated with VIPP1 protein concentration, with higher concentrations favoring wider diameters (Fig. 2e). However, because the rods were likely already formed on the chitin column after DTT-induced cleavage of the fusion protein, variability in loaded protein concentrations, cleavage efficiency, and elution speed prevents the accurate correlation of rod diameter with final preparation concentration. Thus, the comparison between empty and liposome-encapsulating rods must be made within a single VIPP1 prep. We performed this same-preparation comparison by negative stain and found that the addition of PC:PI4P liposomes only had a minor effect on VIPP1 rod diameter (Fig. 2d).

In 55 out of 57 VIPP1 rods analyzed in detail by cryo-ET, we observed helical pitches as seen by the inclination of the striations relative to the long axis of the rod. Rod helical pitches were highly heterogenous, ranging from 43 nm (strongly twisted) to 650 nm (almost parallel), while two rods had parallel striations. Adding to this heterogeneity, VIPP1 rods assembled with either right- or left-handed helical pitches (Fig. 4d; Supplementary Figs. 3 and 4). We also observed discrete points along some rods where diameter and helical pitch abruptly changed, independent of liposome engulfment (arrowheads in Fig. 3b,j). A few rods even switched their handedness at these points (Supplementary Fig. 5).

### The free energy decrease due to interactions between basic amino acids on VIPP1 and PI4P headgroups is sufficient for membrane bending

Our cryo-ET observations indicate that PC:PI4P liposomes are likely sucked into preformed VIPP1 rods (Fig. 3e–k) via specific interactions between the liposome and the inner surface of the VIPP1 rod (Figs. 3h and 4c). Such a mechanism would require dramatic bending of the liposome membrane. To explore whether it is plausible to achieve this bending, we formulated a simple free-energy model of the system. In general, the formation of highly curved lipid bilayers, as observed for the tubular VIPP1-engulfed liposomes, is accompanied by a considerable free-energy penalty, which can be quantitatively represented in a positive (i.e., endergonic) bending free energy *G*_bend_. To estimate *G*_bend_, we modelled a lipid tube as a cylinder of length *l* and radius *r*, resulting in

1 $${G}_{{\rm{bend}}}=\pi {K}_{{\rm{b}}}\frac{l}{r}$$

with *K*_b_ being the membrane bending modulus, which typically amounts to ~20 *kT*^36,37^, where *k* is Boltzmann’s constant and *T* is the absolute temperature. To obtain the bending free energy per lipid molecule ${G}_{{\rm{bend}}}^{{\rm{lip}}}$, Eq. 1 was divided by the number of lipid molecules in the cylinder *n*_lip_ according to

2 $${G}_{{\rm{bend}}}^{{\rm{lip}}}=\frac{{G}_{{\rm{bend}}}}{{n}_{{\rm{lip}}}}=\frac{\pi {K}_{{\rm{b}}}\frac{l}{r}}{\frac{2}{{A}_{{\rm{L}}}}2\pi lr}=\frac{{K}_{{\rm{b}}}{A}_{{\rm{L}}}}{4{r}^{2}}$$

where *A*_*L*_ denotes the area per lipid molecule, which is 0.63 nm^2^ for PC^38^. Using the lipid-tube radii obtained from cryo-ET (Supplementary Fig. 6a), we computed ${G}_{{\rm{bend}}}^{{\rm{lip}}}$ values of 0.07 kJ/mol ± 0.04 kJ/mol (Supplementary Fig. 6b). For liposome engulfment to occur spontaneously, favorable protein–lipid interactions need to exceed the free-energy penalty incurred by membrane bending. We assumed that only PI4P engages in such interactions, as lipid engulfment was found to depend on the presence of PI4P. Since only one bilayer leaflet is available for interactions with the surrounding rod, VIPP1-accessible PI4P makes up 2.5 mol%, or 1/40 • 100 mol%, of the total lipid. Hence, the favorable decrease in free energy per PIP molecule needs to be at least −40 • 0.07 kJ/mol $\approx$ −3 kJ/mol. Importantly, this is considerably smaller than the typical free energy decrease of interactions between basic amino acids and acidic lipids, which is around −6 kJ/mol^39^. Consequently, this simple model suggests that interactions between basic residues on VIPP1 and acidic PI4P headgroups are sufficiently strong to overcome unfavorable membrane bending, thus rationalizing liposome engulfment by VIPP1 rods from a thermodynamic viewpoint.

### VIPP1: a novel PIP-dependent membrane-shaping protein?

Although it has been appreciated for decades that phosphatidylinositols are present in chloroplast membranes^30^, very little is known about their function in this organelle. Only a few PIP-binding chloroplast proteins have been reported. These include the outer envelope-spanning PDV1 and PDV2 proteins that bind to PI4P in the envelope and are involved in chloroplast division^40^, as well as the wheat WKS1 kinase that binds to phosphatidic acid and PIPs via a START domain and phosphorylates a thylakoid-associated ascorbate peroxidase^41^. Although our results indicate that VIPP1 is another PIP-binding chloroplast protein, it is puzzling that phosphatidylinositols do not exist in cyanobacteria^29^, but VIPP1 function is conserved from cyanobacteria to plants. Therefore, either chloroplast VIPP1 has evolved a different lipid specificity, or PIPs mimic an unidentified and rare lipid type, modification, or composition present in both chloroplast and cyanobacterial membranes. In any case, it is noteworthy that PIPs in other cellular membranes serve as targeting sites for proteins with membrane-shaping properties reminiscent of those observed here for VIPP1^42^. One such protein is epsin, which binds to plasma membrane areas enriched in PI(4,5)P2 and deforms the membrane via insertion of an amphipathic helix, facilitating the formation of clathrin-coated vesicles^43^. BAR domain proteins interact with membrane areas enriched in PI(4,5)P2 and phosphatidylserine via cationic residues at the concave surface of their banana-shaped structure to induce membrane curvature^44^. The yeast BAR domain proteins Pil1 and Lsp1 can assemble into long filaments that align their BAR domains on the plasma membrane to form a “half-pipe” static furrow^45,46^. These furrows, termed eisosomes, act as scaffolds that recruit specific proteins and lipids. Many membrane proteins must be localized to eisosomes in order to function optimally^47^. *In vitro*, Pil1 and Lsp1 can tubulate PI(4,5)P2-containing liposomes^45^ in a similar manner to VIPP1.

### What are the implications of our *in vitro* observations for VIPP1 function within the cell?

Analogous to eisosomes formed by Pil1 and Lsp1, VIPP1 rods might play a role in organizing domains in thylakoid membranes at which translocases such as TAT and Sec, as well as integrases like Alb3, are recruited into a lipid environment that is essential for their proper function. This would explain how VIPP1 facilitates protein translocation across, and protein integration into, thylakoid membranes^13,14^ and thereby VIPP1’s role in the biogenesis and repair of thylakoid membrane protein complexes^1,2,7–13^. VIPP1 rods may also play a role in organizing domains in the inner envelope of chloroplasts, giving rise to filamentous and lattice-like structures^4,5^. Such structures might help preserve the integrity of stressed membranes (e.g. under osmotic stress) or might have an analogous function to eisosomes in supporting the function of membrane transporters. Long tubules with the same diameter as VIPP1 rods, referred to as microtubule-like structures, have been observed in the stroma of various plastid types in early electron microscopy studies^22,48–59^. Thus, it is also possible that VIPP1 rods with engulfed lipids exist in the chloroplast stroma with possible functions in lipid transfer or lipid storage.

## Methods

### Expression and purification of VIPP1, tVIPP1 and CGE1

VIPP1 (pMS319), tVIPP1 (pMS388) and CGE1a (pMS300)^22,28^ were produced as fusion proteins in *E*. *coli* ER2566 and purified by chitin affinity chromatography according to the manufacturer’s instructions (New England Biolabs), but including a washing step with 5 mM Mg-ATP to remove potentially bound DnaK^28,60^. Eluted VIPP1, tVIPP1, and CGE1 were concentrated in Amicon Ultra-15 centrifugal filter devices (Millipore) following dialysis as described previously^20^ with the final dialysis buffer containing 50 mM NaCl, 75 mM NaSCN, 20 mM Tris–HCl pH 7.5. Proteins were frozen in liquid nitrogen and stored at −80 °C. Six independent VIPP1 preparations were used in this study: Preps (#1) 0.7 mg/mL; (#2) 0.4 mg/mL; (#3) 0.3 mg/mL; (#4) 0.4 mg/mL; (#5) 0.6 mg/mL; and (#6) 1.5 mg/mL.

### Protein–lipid overlay assay

Protein binding to lipids spotted onto membrane lipid strips was performed according to the manufacturer’s instructions (Echelon Biosciences). Briefly, lipid strips were blocked with PBS-T (0.1% v/v Tween-20) +3% fatty acid-free BSA for 60 min at 22 °C. The lipid strips were then incubated with 0.5 µg/mL purified VIPP1 (Prep #3), tVIPP1 and CGE1 diluted in PBS-T + 3% BSA for 60 min at 22 °C, followed by three wash steps with PBS-T for five minutes each. Membrane-binding of the proteins was analyzed by incubating the lipid strips with antisera against VIPP1^60^ and CGE1^61^ diluted 1:5,000 and 1:3,000, respectively, in PBS-T + 3% BSA for 60 min at 22 °C. An anti-rabbit-HRP secondary antibody (Sigma-Aldrich) was used in a 1:10,000 dilution in PBS-T + 3% BSA for 60 min at 22 °C. After each antibody incubation, the membrane strips were washed with PBS-T for five minutes. Immunodetection was carried out by employing enhanced chemiluminescence (ECL) and a FUSION-FX7 Advance™ imaging system (PEQLAB).

### Liposome preparation

1-palmitoyl-2-oleoyl-*sn*-glycero-3-phospho-(1′-*rac*-glycerol) (PG) and L-α-phosphatidylinositol-4-phosphate (PI4P) from porcine brain were purchased from Genzyme (Cambridge, USA) and Avanti Polar Lipids (Alabaster, USA), respectively. 1-palmitoyl-2-oleoyl-*sn*-glycero-3-phosphocholine (PC) was a kind gift from Lipoid (Ludwigshafen, Germany). Mixtures of PC:PG or PC:PI4P, each at a molar ratio of 95:5, or PC alone, were prepared in 20:9:1 mixtures of chloroform, methanol and water. After evaporation of the solvent under a nitrogen stream and by subsequently applying vacuum in a desiccator, the lipid film was resuspended in aqueous buffer (20 mM HEPES KOH, 80 mM KCl, at pH 8) by vortexing for ~20 min, and the resulting lipid dispersion was subjected to five freeze–thaw cycles. To obtain large unilamellar vesicles (LUVs), the lipid suspension was extruded using a LipoFast extruder (Avestin, Mannheim, Germany) and two stacked polycarbonate membranes having a pore diameter of 100 nm (Whatman, GE Healthcare, Chicago, USA). Vesicles were prepared at 22 °C and stored at 4 °C. Vesicle sizes were determined by dynamic light scattering on a Zetasizer Nano S90 (Malvern Instruments, Worcestershire, UK) equipped with a 633 nm He–Ne laser using a detection angle of 90° at 22 °C. Typical *z*-average vesicle sizes were ~130 nm (PC) and ~120 nm (PC:PG and PC:PI4P).

### Sucrose gradient centrifugation

50 µL of purified VIPP1 proteins (Preps #3 and #4, adjusted to 0.3 mg/mL) were added to 12.5 µL of liposomes (4 mM) containing either PC or PC:PI4P and agitated for 60 min at 37 °C and 400 rpm. The mixture was loaded onto a linear 10–40% sucrose gradient containing 20 mM Hepes (pH 8.0) and 80 mM KCl. Centrifugation was carried out for 3.5 h at 79,000 *g* and 22 °C with a SW 40 Ti rotor. Fourteen 800-µl fractions were collected from which 16 µl of each were loaded on a 12% SDS-polyacrylamide gel.

### Fluorescence anisotropy measurements

VIPP1 (Preps #4, #5, and #6) and liposomes were mixed at 4 °C with protein and lipid concentrations of 0.4 mg/mL and 0.15 mg/mL, respectively, and incubated for 20 min at 22 °C before being transferred to a 3 mm × 3 mm quartz glass cuvette (Hellma Analytics) for measurement. TRF anisotropy measurements were recorded at 22 °C on a FluoFit 300 fluorescence lifetime spectrometer (PicoQuant) using time-correlated single photon counting. The excitation source was a pulsed LED emitting at *λ*_exc_ = 281 nm ± 5.5 nm. Emission was detected at *λ*_em_ = 355 nm ± 5 nm. Fluorescence decays were recorded with horizontal and perpendicular orientations of polarizers in the excitation and emission paths, respectively, each for 5 min. Photons were counted for 100 ns after each pulse in 4000 channels of 25 ps. The measurements were repeated after the addition of Mg^2+^ to a final concentration of 10 mM and an incubation at 22 °C for 20 min. Fluorescence anisotropy decays were fitted by a bi-exponential decay curve with FluoFit (PicoQuant) using data from 3–40 ns after excitation. Two rotational correlation times were obtained, where the fast one, corresponding to segmental tryptophan motions, was essentially constant at values < 0.5 ns, while the slow one, reflecting larger-scale protein motions, was exploited to probe VIPP1 binding to liposomes.

### Preparation of NMR samples and NMR measurements

Liposome composition was verified using ^31^P NMR. To this end, detergent–lipid mixed micelles were prepared by mixing liposomes of PC, PC:PG and PC:PI4P with a 200-fold molar excess of sodium cholate. These small mixed micelles enable sharp ^31^P NMR signals by the phosphate groups of the lipids (Supplementary Fig. 1). Such signals are at distinct spectral positions for different lipid species and are proportional to the amount of lipid in the sample, thus allowing for both identification and quantification^62^. Spectra were recorded at 243 MHz using an Avance 600 NMR spectrometer (Bruker) with 85% (*v*/*v*) H_3_PO_4_ in D_2_O as external reference. We used an inverse-gated ^1^H decoupling sequence to acquire 512–2048 scans with an acquisition time of 1.6 s and a delay of 6 s. Data were multiplied by an exponential function with a line-broadening factor of 1.0 Hz before Fourier transformation.

### Negative staining

5 μL of sample (Fig. 2a–d: VIPP1 Prep #4 alone and mixed with 200 μM 95:5 PC:PG or 95:5 PC:PI4P liposomes; Fig. 2e: VIPP1 Preps #1 and #2) was applied to glow discharged, 200 mesh copper grids (G2200C, Plano GmbH) that had been coated with homemade carbon film. The sample was incubated for 2 min, blotted, washed three times with water, and then stained with 2% uranyl acetate for 30 s. Images were recorded using a FEI Tecnai F20 FEG microscope operated at 200 kV with an FEI Eagle CCD camera, a magnification of 50,000 × (2.21 Å per pixel) and a defocus range of −2 to −5 μm. Measurements of negative stain images were performed with IMOD^63^ and ImageJ^64^ software packages.

### Cryo-electron tomography

Cryo-EM grids were prepared with fresh protein that had not been freeze–thawed. 4 μL of sample (Prep #2 for VIPP1 alone and Prep #4 for VIPP1 mixed with 4 mM 95:5 PC:PI4P liposomes) and 1 μL of 10 nm colloidal gold suspension were applied together to glow discharged, holey carbon-coated copper grids (R 2/1, 200 mesh, Quantifoil Micro Tools, Jena, Germany). Grids were plunge-frozen in a liquid ethane/propane mixture using a Vitrobot Mark 4 (Thermo Fisher, FEI). Blotting chamber conditions were set to 4 °C and 95% humidity, and grids were blotted using blot force 10 and a blot time of 8 s. Grids were stored in liquid nitrogen until usage. Tomograms were acquired on a FEI Titan Krios at 300 kV, using a post-column energy filter (Quantum, Gatan) and a K2 Summit direct electron detector (Gatan) operated in counting mode at 12 frames per second and a calibrated pixel size of 3.42 Å. Tilt series were recorded with SerialEM software^65^ using a bidirectional tilt scheme from 0° to ± 60°, with 2° tilt increments, a defocus range of −5 to −6 μm, and a total accumulated dose of ~100 e−/Å.

### Tomogram reconstruction

Frames from the K2 camera were aligned with MotionCor2^66^ using 3 × 3 patches. Tilt series were aligned with the IMOD software package^63^ using fiducial-based tracking, or image-based patch tracking when not enough 10 nm gold particles were present in the images. Tomograms were reconstructed with weighted back projection.

### Subtomogram averaging

The subtomogram analysis was performed with 4x binned data (pixel size of 1.368 nm). In IMOD, the long axes of VIPP1 rods were roughly defined by manually placing points along the center of each rod. These central points were used to generate a spline representing the rod axis. A tubular grid was then generated as a set of points with a radial distance from the spline. The initial grid spacing was ~5.5 nm, which was chosen to oversample the underlying asymmetric units. Grid points were then used as subtomogram positions, and initial Euler angles were generated from the relative orientation of the particle with respect to the central spline. Subtomograms were extracted with a box size of ~87.6 nm. For rods with larger diameters (e.g., 56 nm and 61 nm), the box size of the subtomograms was ~129 nm. Subtomogram averaging was performed with scripts derived from TOM^67^, AV3^68^ and Dynamo^69^. An initial reference was generated using geometrically-defined Euler angles, which produced a cylindrically averaged tubular section. From there, a six-dimensional search was performed to refine Euler angles and Cartesian shifts, resulting in a low-resolution structure; this reference was used to align the full data set. Initially, half of the rod (defined radially) was masked to perform a local search for ten iterations. From cross-correlation and visual analysis, bad subtomograms were identified and discarded. Alignments converged after four iterations. The averaged structure of the rod after the fourth iteration was used a new reference, and a new cylindrical mask was created. Further averaging was performed for six more iterations. To resolve the averaged structure radially, rather than doing a local search, a global search was performed using the entire rod. This alignment started with the averaged structure after the fourth iteration as a reference and performed six more iterations. These final averaged structures of individual rods were used to measure the geometric parameters plotted in Fig. 4.

### Measurement of VIPP1 subtomogram averages

Geometric parameters were measured for the average of each rod as follows: *Number of striations:* Using UCSF Chimera^70^, the number of striations were counted from the cross-section of the average (see Supplementary Figs. 3 and 4). *Rod diameter and rod-liposome distance:* Rod diameter was measured using radial EM density distribution plots, which were generated by converting the Cartesian volumes to polar cylindrical coordinates and averaging along the azimuthal direction and the rod’s long axis. Diameter was defined as the distance between the outer surfaces of the rod, measured across the rod’s cross-section (as diagrammed in Fig. 4c). Rod-liposome distance was measured as the distance between the center point of the engulfed liposome’s membrane bilayer and the center point of the outer VIPP1 rod’s protein density. *Helical pitch:* The pitch of a helix is defined as the distance along the helical axis (the long axis of the VIPP1 rod) to complete one turn (as diagrammed in Fig. 4d). In other words, pitch is the rise of one full turn. In the Cartesian space volume, two random points were chosen on a single striation of the averaged VIPP1 rod. The helical rise was measured as the distance between the two points parallel to the rod’s long axis. The rotation angle was measured as the angle between the points, projected on the XY-plane. The rise and rotation angle were used to calculate the pitch.

## Supplementary information

Supplemental Figures
