# Supplementary material for: VIPP1 rods engulf membranes containing phosphatidylinositol phosphates
Source: Sci Rep. 2019 Jun 19;9:8725. doi: 10.1038/s41598-019-44259-3 (PMC6584618; doi:10.1038/s41598-019-44259-3)
Supplement: Supplementary file 1 — Supplemental Figures [file 41598_2019_44259_MOESM1_ESM.pdf]

VIPP1 rods engulf membranes containing phosphatidylinositol phosphates

Jasmine Theis[1,*], Tilak Kumar Gupta[2,*], Johannes Klingler[3,*], William Wan[2], Sahradha Albert[2], Sandro Keller[3§], Benjamin D. Engel[2,§], and Michael Schroda[1,§]

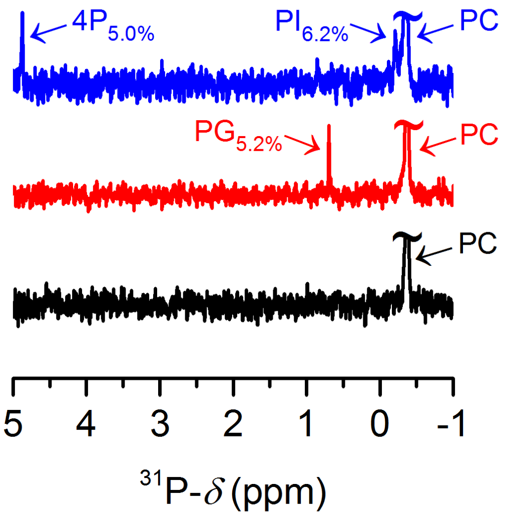

Supplementary Fig. 1: ^{31}P NMR spectra of mixed micelles composed of sodium cholate and PC (black), PC:PG (95:5) (red), or PC:PI4P (95:5) (blue). Signals from PC, PG, and PI4P are near −0.4 ppm, 0.7 ppm, and both −0.2 ppm and 4.9 ppm, respectively. Relative contributions of PG and PI4P to the total ^{31}P signals are indicated.

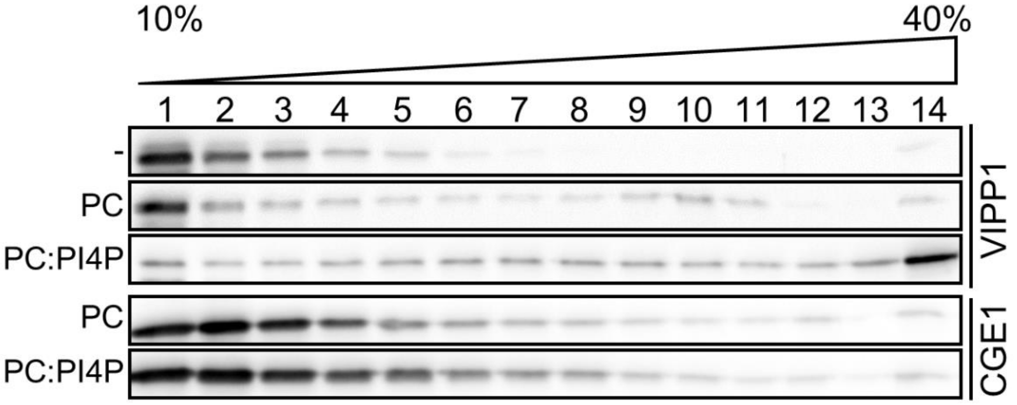

Supplementary Fig. 2: Sucrose density gradient centrifugation. Recombinant VIPP1 and CGE1 were incubated with liposomes composed of PC and PC:PI4P (95:5), loaded onto a 10–40% sucrose gradient and centrifuged for 3.5 h at 79,000 g. 14 fractions (not including the pellet fraction) were collected and immunodetected for the specified proteins.

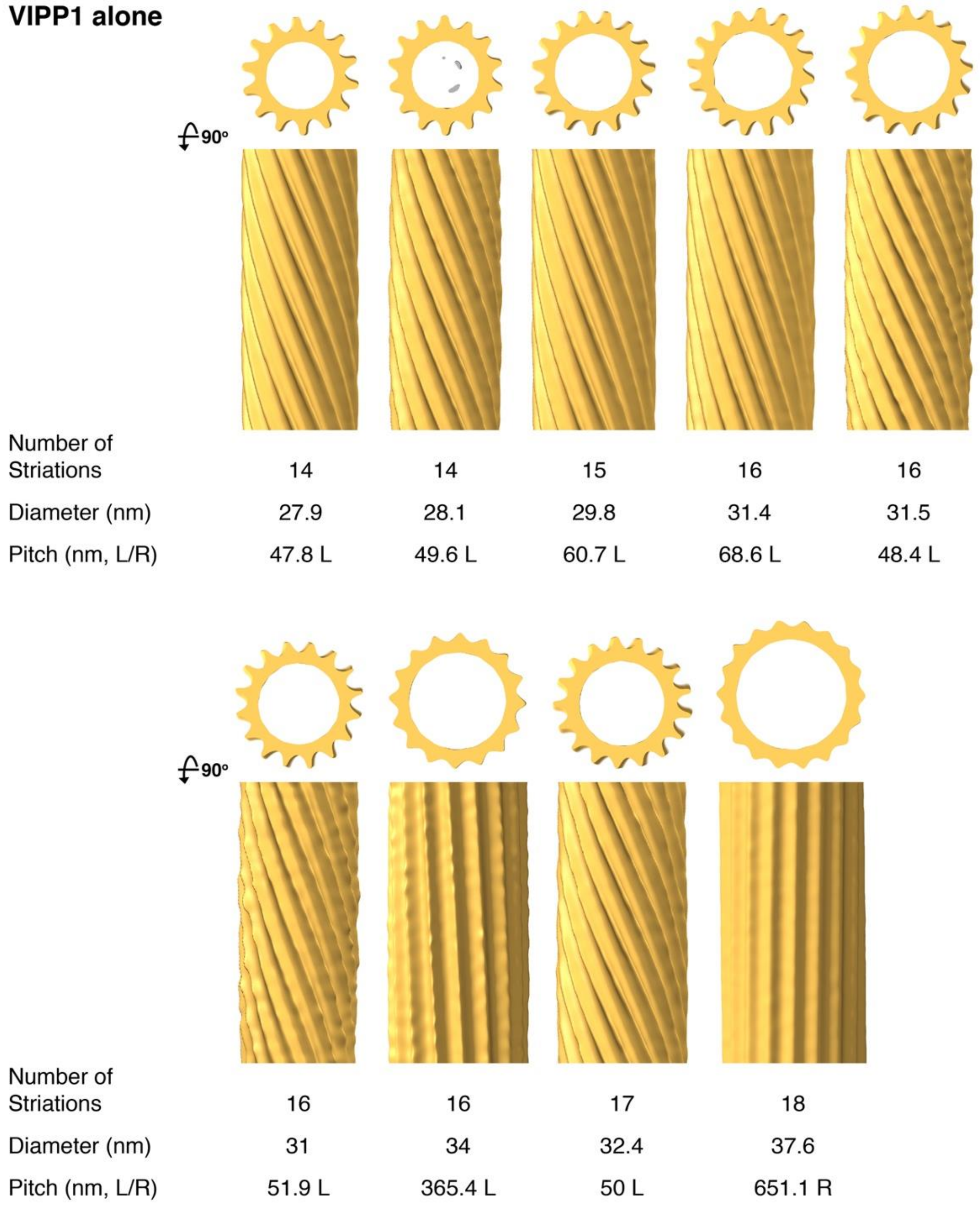

Supplementary Fig. 3: Additional subtomogram averages of VIPP1 rods alone (yellow). Top: cross-sections through averages, bottom: longitudinal views of the rod outer surfaces. Rods are shown to scale. Parameters quantified in Fig. 4b-d are displayed for each individual rod.

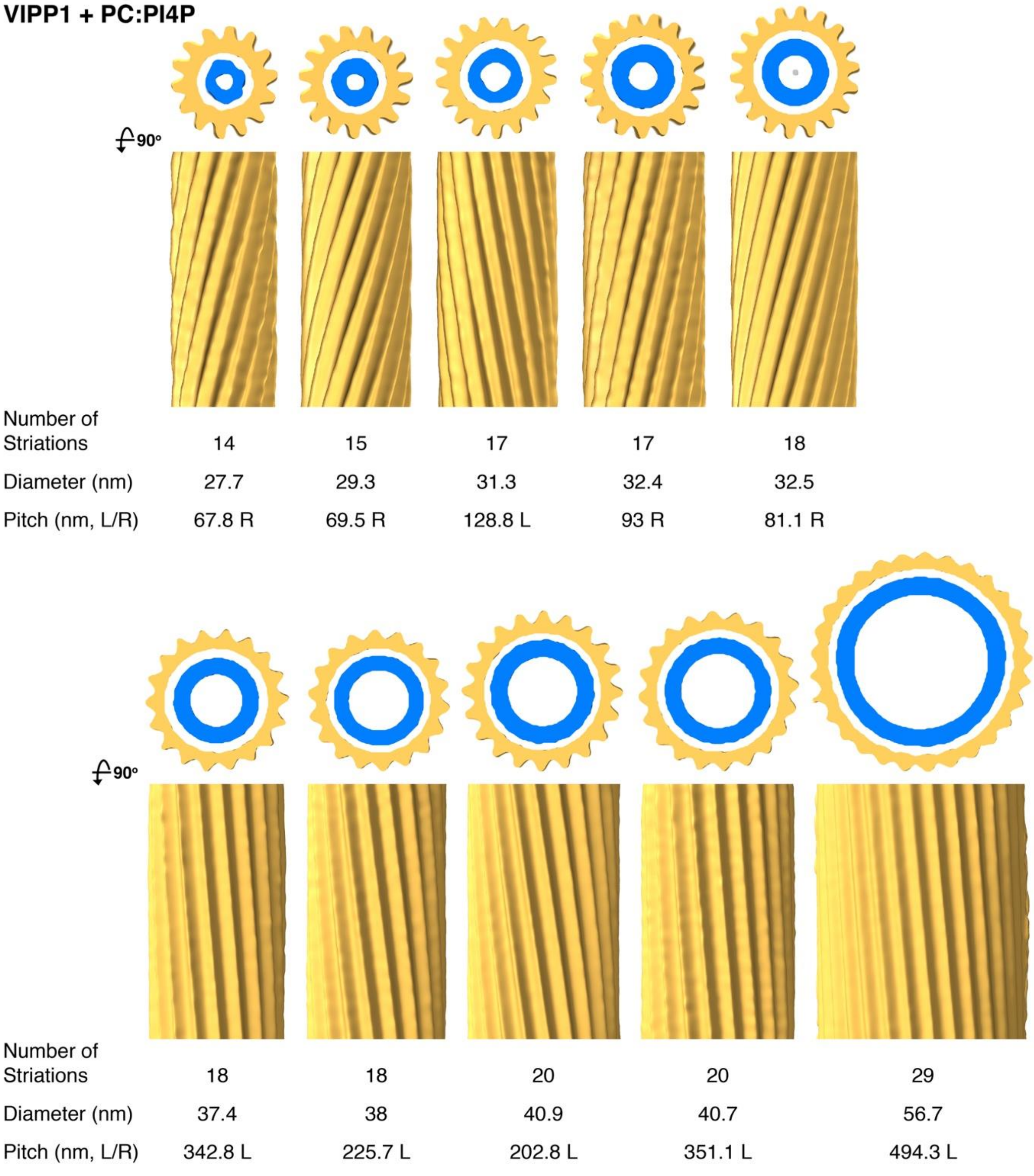

Supplementary Fig. 4: Additional subtomogram averages of VIPP1 rods (yellow) incubated with PI4P-containing liposomes (blue). Top: cross-sections through averages showing the engulfed liposomes, bottom: longitudinal views of the rod outer surfaces. Rods are shown to scale. Parameters quantified in Fig. 4b-d are displayed for each individual rod.

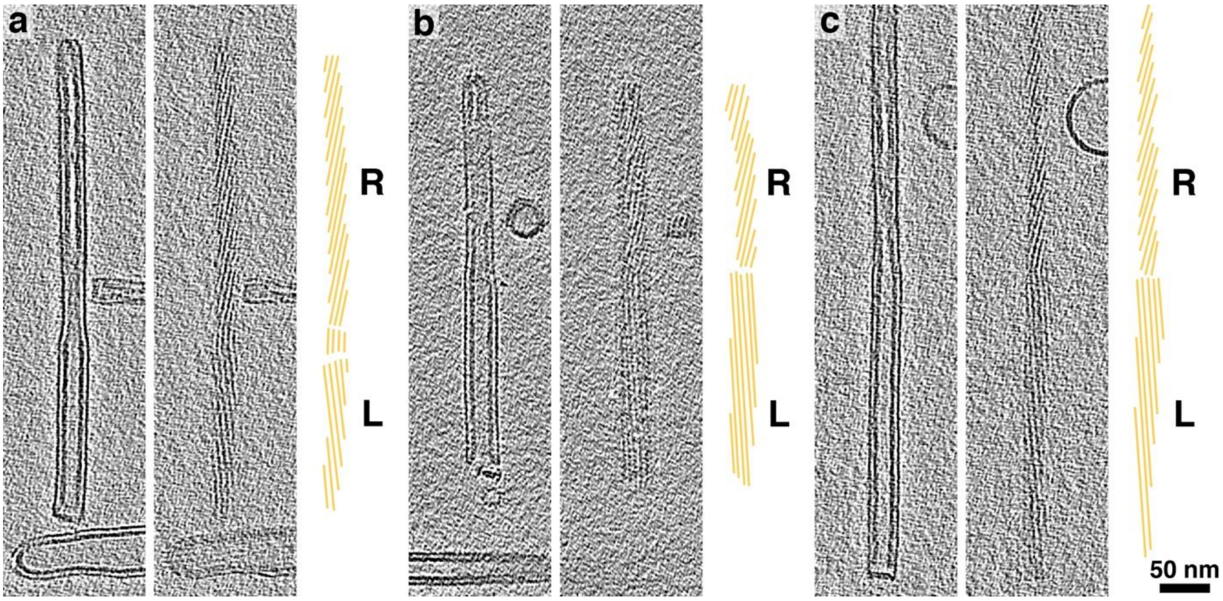

Supplementary Fig. 5: a-c, Three VIPP1 rods that switch from a right-handed helix (R) to a left-handed helix (L) at discrete points along their lengths. For each panel, the left image is a slice from the tomogram showing a central longitudinal section through the VIPP1 rod (with encapsulation of PC:PI4P liposomes), the middle image is a slice from the tomogram showing the top surface of the rod, and the right image shows lines that were draw along each rod’s striations (yellow) to illustrate the helical pitch. These examples serve as a control to visually confirm that VIPP1 rods adopt both right- and left-handed helical pitches.

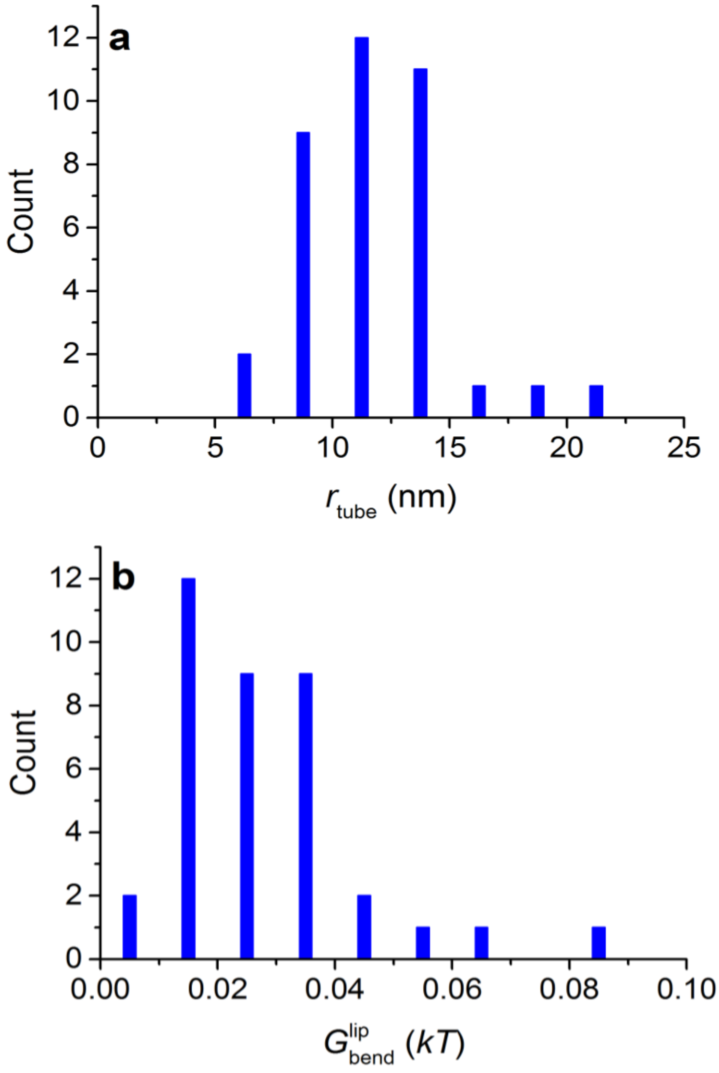

Supplementary Fig. 6: Distributions of **a**, lipid tube radii and **b**, calculated bending free energies per lipid molecule.
